# The Dawning of the Ethics of Environmental Robots

**DOI:** 10.1007/s11948-017-9990-3

**Published:** 2017-10-23

**Authors:** Aimee van Wynsberghe, Justin Donhauser

**Affiliations:** 1 Technical University of Delft, Jaffalaan 5, 2628 BX Delft, Netherlands; 2 Rotman Institute of Philosophy, Western University, 2150 Stevenson Hall, London, ON N6A 5B8 Canada

**Keywords:** Robot ethics, Environmental robotics, Ecological robots, Ethics and technology, Environmental engineering

## Abstract

Environmental scientists and engineers have been exploring research and monitoring applications of robotics, as well as exploring ways of integrating robotics into ecosystems to aid in responses to accelerating environmental, climatic, and biodiversity changes. These emerging applications of robots and other autonomous technologies present novel ethical and practical challenges. Yet, the critical applications of robots for environmental research, engineering, protection and remediation have received next to no attention in the ethics of robotics literature to date. This paper seeks to fill that void, and promote the study of environmental robotics. It provides key resources for further critical examination of the issues environmental robots present by explaining and differentiating the sorts of environmental robotics that exist to date and identifying unique conceptual, ethical, and practical issues they present.

## Introduction

The robotics revolution is upon us. The *Executive Summary of the International Federation for Robotics* shows substantive increases in robot sales across most every sector from 1 year to the next; including a twenty-five percent increase in the total number of service robots sold in 2015 alone (IFR Press). In 2017, at the time of writing this paper, ethical and societal reflections surrounding emerging robotics technologies have largely focused on impacts they may have on labor markets. Certain ethical concerns are also on the forefront of the minds of designers, journalists, academics, policy-makers, and consumers—including issues regarding the level of autonomy robots should have to more applied issues like the impact robotic technologies may have on public safety and personal privacy. Indeed, issues like these receive continued attention in ethics and technology literature (see, e.g., Asaro 2006; Capurro 2009; Lin et al. 2011; van Wynsberghe 2016). There is, nevertheless, a massive and overlooked lacuna in discussions about the ethics of robotics; their critical applications for environmental research, engineering, and remediation have received next to no attention in the roboethics literature to date (Sullins 2011).

Of course, increased use of robots in general can be evaluated as an environmental burden when considering: the materials needed (minerals and hardware); where and by whom the processes for degradation of the robots will happen (the current processes for smart phones and other technological devices occurs in under developed countries by children and other vulnerable demographics); and, the regulation for these devices. It is also clear that robots could reduce certain human impacts on the environment by helping monitor pollution outputs and/or endangered species. Yet, as accelerating environmental, climatic, and biodiversity changes push scientists, engineers, and various stakeholders to explore new environmental applications, and even functional integrations of robotics technologies into ecological systems, we must confront a multitude of novel ethical challenges. And it is not so clear how to understand or evaluate the challenges presented by emerging *environmental* applications of robots because the very nature of these technologies remains unclear. Answering two simple questions is thus crucial for critical discussion of the ethics of environmental robotics to progress and for environmental robotics research, industries, and design culture to move forward in a responsible manner: What are the distinct kinds of environmental robots that exist? And what unique ethical and practical challenges do they present? This paper addresses these questions as follows.

After discussing key motivations for this project and providing some theoretical background, a foundational taxonomy of environmental robotics is proposed. In sum, it makes substantive distinctions between robots used in environmental research, those designed for specialized environmental research applications, and those used to play functional ecological roles in natural and engineered environments. For reasons fleshed out below, these are distinguished as ‘robots-in-ecology,’ ‘robots-for-ecology,’ and ‘ecologically-functional-robots’ (henceforth, ecobots) respectively. Following an explanation of these classifications, the proposed scheme is clarified and reinforced through discussion of prime examples of each sort of environmental robotics technology. Against that background, this paper then finishes by identifying key ethical issues presented by these emerging technologies, which are offer as subjects for crucially important follow-up work that can build on the resources offered herein. Accordingly, this paper’s overarching aims are to:

- Raise awareness of the different kinds of environmental robotics technologies that exist to date;
- Promote further exploration of environmental robotics technologies, their potential valuable applications, and the ethical, practical, and broadly sociopolitical challenges they may present;
- And provide theoretical resources to facilitate and guide more pointed analyses of the challenges presented by emerging environmental robotics technologies.

### Why an Ethics of Environmental Robotics is Needed

Roboethicist John Sullins pointed out in 2011 that the rise of the roboethicist comes in tandem with the rise of robotics. This roboethicist, Sullins says, “is tasked not only with critiquing the attempts of robot engineers to achieve the integration of these machines into our life world, but also, and more importantly, with suggesting means of achieving better results than what is presently on offer” (pg 233). In the same paper, he also points out that “there is no green robotics movement and we should push for this to be developed” (pg 237). Sullins’ call is in effect a mainspring for this paper.

Around the globe, unprecedented and accelerating environmental changes are occurring; including climate change impacts and biodiversity losses that pose threats to valuable resources, economies, and public safety. Hundreds of countries have explicitly recognized the urgency of these problems and have begun developing mitigation and adaption responses to the now unavoidable impacts of human driven, “anthropogenic,” environmental change (see UNFCCC 2013; UNFCCC 2015). The associated global sociopolitical culture—with its wholesale commitment to pushing forward a green economy and the pursuit of sustainable technologies and practices—suggests that environmental robotics technologies are likely to be explored more and more. The above noted expansion of more general interest in robotics technologies reinforces this conclusion. Additionally, many environmental robots already exist (as discussed below) and the potential for their ever-more rapid development and use itself motivates consideration of ethical and practical concerns associated with such technologies. The absence of literature on the ethics of environmental robotics is also itself motivation. Further still, such technologies and concerns about them demand attention because there are incentives to pursuing robotics solutions to mounting environmental issues.

One big incentive is that technology-based responses to mounting environmental problems may outstrip demonstrably slow and unpredictable sociopolitical reactions. The sluggishness and instability of large-scale sociopolitical solutions is highlighted by the decades of negotiating that *United Nations Framework Convention on Climate Change* (UNFCCC) agreement drafted at the *Paris Convention* (COP21). The agreement lays a foundation for the most massive institutional response ever to the impacts of human activity on Earth, as 195 parties initially signed the agreement and committed to contribute to the realization of international “climate action plans”. This is certainly an impressive manifestation of the global “green” culture just mentioned. Yet, notably, it took over 20 years of convention meetings just to reach agreement on courses of action; and the international cooperative actions that agreement outlines are sensitive not only to uncertainties about environmental factors but also to unpredictable social, political, and economic turbulence.

Indeed, during the writing of this paper, U.S. President, Donald Trump, abruptly withdrew participation in the Paris Agreement; forcing the U.S. to join only two other parties to the agreement (Syria and Nicaragua) who have failed to commit (see Light 2017). The potential ripples of this decision are yet to be felt, but one thing is for sure. Given the sensitivity of national and international initiatives and institutions to such political maneuvers, it behooves companies, academics, regional and local governments, and NGOs to pursue options for addressing environmental issues on their own steam. Trump’s decision appears to have made this clear to many; as companies, States, and Countries were voicing this sentiment with their plans to double-down on their previous commitments to realizing the measures of the Paris Agreement in the hours and days following the announcement of the decision. Such sentiments, coupled with rising sales, production and availability of robotics in general, also suggest that environmental robotics will (and arguably should) play an increasingly prominent role in environmental protection and resource management in years to come. As the discussion below will show, it is simply becoming easier and easier to use robots to monitor environmental conditions and hazards and to apply robots to aid in addressing certain environmental issues.^1^

As a final bit of background, it is also instructive to explain the motivations for highlighting uses of robotics for *ecological* research and engineering in the discussion below. This is motivated primarily by the fact that analyzing applications of robots for ecological research and engineering is especially crucial for providing resources to expand the technical field of environmental robotics. One reason this is so is that ecology is the keystone science that provides the sorts of causal network thinking that undergirds contemporary environmentalism and environmental ethics. Indeed, leading environmental advisory organizations look to ecological theory and research and engineering practices as objective guides for environmental policy and management decisions at all scales. For example, among many others, ecological considerations are a central component of efforts including the UNFCCC and *the STRATEGY Framework EU project* (UNFCCC 2015; EURANOS 2006; see also, Donhauser 2016, 2017). Hence, as the science of complex biophysical dynamics of direct relevance to such public policy and resource management decision-making, “ecology is only one small step away from urgent political, ethical, and management decisions about how best to live in an apparently increasingly-fragile environment” (Colyvan et al. 2009, p. 1). Accordingly, the following discussion of environmental robotics and sub-classes of ecological robotics will provide resources for academics and industry leaders in robotics and roboethics, and may have significant implications for existing environmental policy institutions which have increasingly focused on articulating and addressing the ethical dimensions of ecological remediation and engineering.

A related reason for highlighting *ecological* research and engineering applications of robots is that promoting awareness and understanding of the kinds of functional ecological roles that emerging robotics technologies can play could lead to the development of crucially valuable new defenses against mounting unprecedented environmental changes and their impacts on valued resources. Yet, as a final background caveat, one should take note that ‘ecology’ is used somewhat loosely in the proposed classification scheme below; primarily to avoid using more cumbersome labels like ‘robots-used-in-environmental-research.’ So, while it is important to analyze uses of robotics in *ecological* research and engineering for the reasons just outlined, one should also recognize that the proposed classifications of kinds of environmental robots and associated ethical issues extend to environmental robotics technologies that may see uses in domains that some may not consider to fall within ecology-proper.

### First, What Counts as a Robot?

To see their potential advantages, and to do so efficiently and ethically, academics, industry leaders, NGOs, and policy makers need to first understand the various types of robotics technologies that can be used for environmental purposes as well as the unique challenges they may present. The first step in getting clear about the nature and kinds of environmental robot technologies is getting clear about what technologies count as robots. When considering the notion of ‘environmental robotics’ one may initially conjure images of humanoid automatons cleaning up environmental toxins or planting trees. And below one will see that some environmental robots in fact operate like this to some extent. Yet, the discussion below also shows that many clearly do not. In fact, generally speaking, most robots that exist to date do not fit that futuristic artificial human image. Interestingly though, that futuristic prototype conception is useful, as one can hone in on the essential nature of ‘robot’ by digression from the pop-culture automaton image.

That prototype conception was introduced by Czech playwright Karel Čapek in his 1920 play *Rossums’s Universal Robot*; about the manufacturing of artificial human beings to do the work of humans. Čapek adapted the word ‘robot’ from the old Slavonic word, *robota*, meaning essentially “forced servitude” or “slave.” Much later, the creator of ‘robotics’ (the study of robots), Isaac Asimov gave us the “Three Laws of Robotics.” According to Asimov a robot is essentially ‘machine + computer,’ as he says: “a robot is a computerized machine that is capable of performing tasks of a kind that are too complex for any living mind other than that of a man, and of a kind that no non-computerized machine is capable of performing” (1990, 2). Čapek’s and Asimov’s conceptions both have a clear view of robots being human-like machines, mechanical metal automatons, able to function autonomously via a central brain-like control system.

Currently these imagined robots (functioning metal helpers that look like humans) do not really exist. With the exception of a few humanoid robots explored commercially—e.g. the Pepper or Nao robots of Softbank, and many “sex robots” such as those of True Companion or Abyss Creations—the majority of commercially available robots are far more machine-like than human (e.g. agriculture robots, drones, surgical robots). What’s more, it appears that many features of the Čapek/Asimov conception are superficial and unessential to robots upon reflection.

For starters, there is no principled reason to think it is essential to robots that they are human-like. Surely, robot horses or birds or fish should not be counted out as robots just because they are not created in the human image. Moreover, there seems no good reason to rule out technologies on the basis of the materials from which they are made. Plastic and wooden robots it seems would be no less robot than a wooden bike is less of a bike for being made from historically unconventional materials. Accordingly, it seems even organic materials, like synthetic tissues, could be used in the production of bona fide robots. And finally there is no reason to maintain that robots must be capable of things that are “too complex for any living mind” apart from humans; indeed a robot that could fetch one’s slippers would not fail to be a robot just because it only achieved the capabilities of a service dog.

In line with these thoughts and the ever-changing popular conception of what robots can be, as more and more different sorts become realities, popular conception of what a robot is has shifted more toward defining them in terms of their origin and functionality. That is, defining ‘robot’ not in terms of ‘how they emulate humans but differ from us in their constitution’ but in terms of their being man-made machines that can be made to carry out specified tasks autonomously. Thus, the features from the Čapek/Asimov conception that appear essential are that robots are intentionally created technologies that can autonomously carry out specified jobs upon command. Robots are synthetic slaves, and environmental robots are those that can carry out jobs for environmental research, engineering, and protection.

A further general distinction made in the ethics and technology literature that is helpful in narrowing down what counts as an environmental robot is the distinction between industrial robots and service robots. In sum, the former do their work in a factory and the latter function outside factory settings. Environmental robots (at least most that exist) fall into the latter category; making the study of environmental robotics the study of environmental service robots. As such, they adhere to the definition of service robots from the *International Organization for Standardization*, according to which service robots are those that: “perform useful tasks for humans or equipment excluding industrial automation applications” and can (and do) operate with varying degrees of autonomy ranging “from partial autonomy—including human–robot interaction—to full autonomy—without active human–robot intervention” (ISO 8373). Specifically, environmental robots are those service robots that “perform useful tasks” in the service of environmental research, engineering, protection and remediation with some level of autonomy. To illuminate the ways in which robots can serve such “environmental” functions and suss out the ethical issues that different kinds of environmental robots present, we will now lay out the promised taxonomy of environmental robotics in general terms and then discuss exemplary instances, and subspecies, of each major kind of environmental robot in the proposed scheme.

### A Guiding Taxonomy for Environmental Robot Ethics

**Fig. 1:**
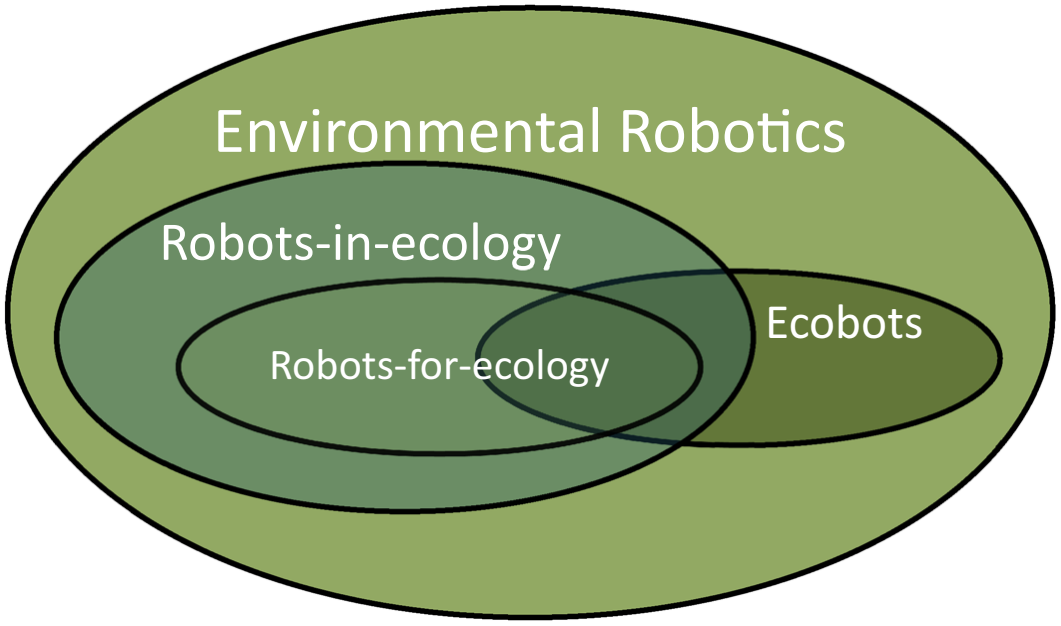
Conceptual map of the proposed taxonomy of environmental robotics

In line with the discussion so far, the study of environmental robotics is here taken to encompass the study of all robots that aid in environmental research, engineering, protection or remediation. Central to this domain, is the study of service robots used and/or designed to perform functions in the service of such endeavors. Yet, the broader domain of environmental robotics also includes consideration of environmental impacts of robotics technologies whose primary functions may be other than serving environmental research and protection aims. This is because the environmental values and impacts of robots can be realized in a multitude of ways. A robot may carry out a role that serves a benefit to the environment or research of it or it may factor into environmental protection concerns by being made from materials that do not present the same harm to the environment as more traditional materials. Accordingly, we propose the following functional classes of environmental robots, and illustrate their overlap in Fig. 1.

We propose substantive distinctions between robots used in environmental research, those designed for specialized environmental research applications, and those used to play functional ecological roles in natural and engineered environments. Bearing in mind the aforementioned emphasis of ecological research and use of ‘ecology’ to connote environmental research more generally for linguistic economy, these are distinguished as ‘robots-*in*-ecology,’ ‘robots-*for*-ecology,’ and ‘ecobots’ (ecologically-functional-robots).

Robots-in-ecology are robot technologies used for environmental research applications; including uses of general robotics technologies for such research. For example, below various uses of drones, or unmanned aerial vehicles (UAVs) are discussed, as well as similar technologies for environmental monitoring and observation of numerous sensitive species. As is indicated in Fig. 1, the name robots-*for*-ecology denotes a subclass of robots-in-ecology that are specifically designed to carry out, usually tedious (e.g. repetitive) or difficult, *research*-*specific tasks* that they can accomplish more efficiently than human researchers. So, whereas environmental monitoring with UAVs and similar technologies is simply an application of a general robotics technology, below we will discuss several kinds of robots-*for*-ecology that have been designed, programmed, or somehow retooled specifically to accomplish specialized environmental research tasks. Then *ecobots* are ecologically functional robots. Although they can be used for research as well, and can include robots-in-ecology that also exhibit ecological functionality, ecobots accomplish their tasks; that is, either by playing some functional ecological role (e.g. serving as a proxy predator) or by augmenting ecological functioning (e.g. enhancing ecosystem services) via autonomous behaviors or controls of key environmental variables. To be sure, none of these categories are exclusive; the overlapping and non-overlapping areas depicted in Fig. 1 represent the possible singular or multiple uses and functions of robots-in-ecology, robots-for-ecology, and ecobots.

Before delving into examination of exemplary kinds of robot technologies that fall into each of the proposed categories, it is instructive to briefly pause to notice the area outside the domain of either robots-in-ecology and ecobots in Fig. 1. This represents the inclusion of different kinds of robots within the study of environmental robotics whose primary use and design is neither to research the environment nor serve ecological roles but which have environmental and ecological impacts. For example, this space includes technologies that may have notable impacts by being made from “environmentally friendly” materials. It would also include those robots whose primary intended use may not be to impact the environment but which may have significant environmental impacts nonetheless—like agricultural robots and robots used to explore new environments inaccessible to human researchers (see, e.g., Aravind et al. 2017; Townsend et al. 2014; Yaghoubi et al. 2013; Yoshida et al. 2016). Here we will leave the consideration of such technologies at this brief mention and now turn to better differentiating and clarifying the nature of robots-in-ecology, robots-for-ecology, and ecobots through critical examination of exemplary kinds and uses of such robots to date.

#### Robots-in-Ecology

Robots-in-ecology include common robotic platforms such as “drones” and “rovers.”^2^ Choi-Fitzpatrick (2014) notes that UAVs are:

[U]sed in a number of environmental areas, including change mapping (i.e. river erosion, deforestation, and urban expansion); disaster risk management and mitigation (assessing natural disaster risk and monitoring fires, volcanoes, and landslides); monitoring illegal activity, including banned hunting, fishing, and trade; and monitoring other natural factors like migration, levels of endangered species, and foliation. (p. 24).

UAVs have been demonstrably useful for monitoring polluting industries in China, monitoring illegal logging in Brazil, and monitoring poaching in national parks in Kenya. And correlative studies show that such monitoring can reduce such violations and irresponsible practices, among others (e.g. large scale corporate violations) by up to 96% (ibid., pp. 24–25). Within ecology, and applied environmental sciences more broadly, UAVs are also most commonly used for monitoring (see Ivoševic et al. 2015). Yet, here their role is not primarily one of identifying environmental problem sources, but one of enabling safer, more efficient, and more ethical research.

Dunbabin and Marques (2012) argue that fairly recent catastrophic events highlighted these benefits of utilizing UAVs and hastened their wide-spread use in research in recent years. In particular, in the preface to their insightful historical survey of monitoring robots, they contend that media attention to the 2010 eruption of Eyjafjallajökull, the massive oil spill in the Gulf of Mexico from the Deepwater Horizon platform, and the 2011 earthquake and tsunami in Japan, jointly underscored that an “obvious advantage of utilizing robotics in environmental sciences is that they allow the monitoring and sampling of events that are too dangerous or impossible for humans to undertake” (20). Though one may debate whether those particular events in fact inspired the subsequent explosion of applications of UAVs in critical research applications, clearly their increased availability and usage hastened a major shift in ecological research—which has seen the “dawning of drone ecology” due to their abilities to enable more efficient and ethical research than is otherwise possible (Koh & Wich 2012).

UAVs have proven especially useful for approaching sensitive species in relatively inaccessible areas by allowing researchers to observe and monitor organisms and populations with minimal to negligible stress on the subjects of study. For example, Vas et al. (2015) document hundreds of trials in which they were able to approach mallards (*Anas platyrhynchos*), wild flamingos (*Phoenicopterus roseus*) and common greenshanks (*Tringa nebularia*) within four meters without affecting the birds at all eighty percent of the time (cf. Hodgson et al. 2016). Yet, UAVs are not a panacea, as they can equally cause stresses to certain species. Ditmer et al. (2015), for example, report documented stress responses in American black bears (*Ursus americanus*) and big horn sheep (*Ovis canadensis*); though they too acknowledge that UAVs have been crucially useful for monitoring endangered rhinoceros (*Diceros bicornis* and *Ceratotherium simum*) and deterring poaching.

Where drones cannot go often terrestrial and aquatic rovers can, and, despite their relevantly similar pitfalls, have also enabled advances in research efficiency and ethics similar to UAVs. For example, to reduce the “large and long-lasting increases in stress hor-mones” associated with “human approaches and manipulations” researcher have used *autonomous ground vehicles* (AGVs) to monitor populations of endangered king penguins (*Aptenodytes patagonicus*) (Le Maho et al. 2014, p. 1). As with UAV research on sensitive and difficulty located birds, this research shows that approaches by AGVs produce a significantly lower stress response (e.g. elevated heart rate) than approaches by human researchers (ibid., p. 2).

Although they too can have applications for reducing human interference as an artifact of research practices, *autonoumous underwater vehicles* (AUVs) were among the first sorts of robots to be widely used to augment research potential (Dunbabin and Marques 2012, p. 25; cf. Whitcomb 2000). AUVs have enabled the exploration of environments and species at aquatic depths (see Yoerger et al. 2000). Equally, they have enabled explorations in complex underwater situations that are exceedingly dangerous, and sometimes practically impossible, for human researchers to go. For instance, AUVs have enabled explorations under exceedingly long arctic ice sheets and tracking of aquatic predators (including numerous species of sharks for example) (see Wadhams et al. 2006; Clark et al. 2013).

As technologies continue to develop and converge with one another, another interesting application of robots entails using them to aid in biologging, which is “logging and/or relaying data about an animal’s movements, behavior, physiology, and/or environment” (Rutz and Hays 2009). Whereas video monitoring with a UAV is simply one application of a general robotics technology, similar robot technologies can serve as robots-for-ecology by being designed, programmed, or somehow retooled to accomplish more specialized tasks. For example, an existing, general, rover technology that has been retooled to perform a specialized repetitive research task was discussed above; with the AVGs that monitor king penguin numbers and movements by roving around and continuously remotely “biologging” data from tags implanted within individual penguins. A similar example is pollution-tracking robots developed by scientists at the University of Singapore, which look like realistic swans but contain water-quality-monitoring apparatuses; and “swim” around autonomously and log data remotely and systematically return to charging stations when they require recharging (Coxworth 2015).

#### Robots-for-Ecology

Robots-*for*-ecology are here conceived as those service robots used in environmental research that are invented and designed for the express purpose of carrying out more highly specialized research tasks with maximum efficiency. A good example of a robot designed to do a difficult specialized task is ‘Treebot’(Lam and Xu 2012). Its inventors note that, at the time of its introduction—at the Robotics and Automation (ICRA), 2011 IEEE International Conference—was the “world’s lightest, smallest and most flexible tree-climbing robot” designed to overcome the limitations of existing tree-climbing robots and maximize efficiency for “tree inspection, maintenance, pest control and monitor[ing] arboreal environment[s] for ecological research” (ibid., p. 140).

Other examples are found among the rapidly expanding array of bio-mimicking robots and robot groups (including various “robot swarms” discussed below) that are designed to fill specific functional research niches with maximum efficiency. Consider, for instance, robots that mimic bacterial locomotion; variations of which have been in development for over a decade now (Dhariwal et al. 2004; see also Hart and Martinez 2006 and Rundel et al. 2009). By mimicking bacterial response to the presence of chemical concentrations, i.e. bacterial chemotaxic behaviors, such robots can play invaluable roles in locating and monitoring chemical sources and tracking chemical gradients in an endless array of environments and for all sorts of environmental research purposes. Notably, uses of many robots-for-ecology cross a threshold such that they have robots functioning as what can be distinguished as *ecobots*. Case in point, biomimicking technologies can easily influence ecological functionality when introduced to natural environments simply by doing the mimicking they are designed to do. And treebots and similar technologies too can easily have ecological impacts if deployed to do things like removal of “pest” or disease species.

#### Ecobots

Two autonomous technologies designed specifically to serve as ecobots have been developed in cooperation with the not-for-profit RSE. The first is the COTSbot, developed by Queensland University of Technology researcher Matt Dunbabin; an AUV that autonomously seeks out the predatory crown-of-thorn starfish (COTS) using visual recognition technology and destroys those predators by injecting them with a toxin (Today’s Eco-robots). The other is the Lionfish Project, which seeks to implement a similar ecobot that will target lionfish. As a highly efficient apex predator that is not culled by other marine species, due to their eighteen venomous spines, “a single lionfish can reduce the fish biomass on a reef by 80% in just 1 month” (Lionfish Project). Since lionfish are currently at seventeen times their historic population level in the Atlantic, the Lionfish Project may produce ecobots responsible for saving the Earth’s coral reefs in our day. Likewise, many sorts of bio-mimicking robots are being explored to serve as defenses against mounting unprecedented environmental changes and their impacts on valued resources.

For instance, researchers at Harvard’s Wyss Institute continue to develop yet another sort of ecobot: “programmable robot swarms.” As the Institute’s website explains:

[A] hive “operating system” could let a user program colonies of robots to perform complex tasks in natural environments such as land, air, and sea. Flying microrobots could be instructed to pollinate a field, or—inspired by termites—an autonomous robot construction team could be programmed to build 3D structures and traversable surfaces, to stack sandbags along vulnerable coastlines before a hurricane or to lay our barriers around toxic chemical spills. (Programmable Robot Swarms).

The possibilities seem endless, as one can easily imagine how tiny robot swarms could systematically repair environmental damages (e.g. by repairing coral or trees) and mitigate threats (e.g. by removing invasive species or removing contaminants).

Other kinds of existing ecobots are not as “futuristic” as the examples above, and have emerged not through applications of robotics to carry out or mimic ecological tasks, but through attempts to repurpose natural ecological functions to aid in environmental protection and remediation. The most basic, widely employed, and historied, kind of ecobots of this sort have their origins in uses of plants for ‘phytoremediation.’ ‘Phytoremediation’ uses plants, and rhizosphere microorganisms associated with them, to remove, chemically stabilize, or contain toxins in soil, ground and surface water, or parts of the atmosphere (Susarla et al. 2002). In parts of the Middle East and Europe, forms of phytoremediation have been implemented for centuries to protect streams from agricultural contamination (Adams et al. 2000). Phytoremediation has gained popularity worldwide over the past three decades, and pytoremediation techniques are increasingly used to clean up classes of contaminants including petroleum hydrocarbons, chlorinated solvents (e.g. TCE), pesticides, explosives, and heavy metals.

#### Pushing the Boundaries of ‘Robot’

One may be inclined to object that plants used for environmental remediation do not constitute robots, since this consideration certainly goes against a traditional conception of ‘robot.’ Yet, given the essential features of robots presented in the above discussion of issues with the traditional Čapek/Asimov conception above, it appears that the boundaries of what robots can be, ought to be expanded—at least for considerations with the domain of environmental robotics. Recall, the ISO definition of a service robot only partially echoes the Čapek/Asimov conception, in specifying that robots must perform useful tasks for humans and command and must also have some degree of autonomy. Notably, engineered plants and biofilms can and do “take commands.” For instance, they can be made to act more quickly or slowly or cease by changing light and nutrient ques. If one were to feed trees more nitrogen, they suck up contaminated water faster; if one wanted them to stop she could block their access to UV light. Those are commands and the plants respond autonomously via their central control mechanisms.

If robots are essentially ‘intentionally created technologies that can autonomously carry out specified jobs upon command’ and ecobots are ‘ecologically functional robots,’ then more organic technologies can surely be ecobots. Indeed, even repurposed natural plants are mechanical since they operate via biochemical mechanisms and perform mechanical functions. More commonly nowadays such things are even man-made, since they are either directly genetically engineered or engineered through selective breeding for traits. And many such technologies have been used to perform human remediation functions, like physical labor to remove contaminants, and are increasingly incorporating both synthetic and engineered-organic systems to serve as ecobots.

#### Back to Considering More Kinds of Ecobots

Whatever one’s inclination on the issue of whether repurposed organic things can be considered robots, it is clear that more contemporary phytoremediation, and associated, autonomous technologies fall more squarely under a standard conception of robots since they are engineered and intentionally designed to perform specific autonomous service functions; for example, “hybrid” macroplants are commonly engineered to maximize their remediative functionality (cf. Dickmann and Stuart 1983; Zalesney et al. 2005). In another instance, genetically engineered hybrid trees (hybrids of poplar or willow species for instance) are cloned via crosspollination of phenotypic variants that are fast growing but lack hardiness and others that are hardy but slow growing. Thusly, macroplants are synthetically designed and engineered to survive and grow exceedingly quickly in a diverse range of conditions, and transpire *a lot* of water—often growing several meters per year and transpiring upwards of 100 L of contaminated water per day—compared to naturally occurring species (see Best et al. 1997; Burken and Schnoor 1998; Gordon et al. 1998; Vangronsveld et al. 2009; Succuro et al. 2009; Tripathi et al. 2008; Tanaka et al. 2007). Among others, the mechanisms by which ecobot plants carry out phytoremediative processes through autonomous functions include:

- “hydraulic control” whereby the spread of waterborne contaminants is limited through plant uptake and evapotranspiration;
- “phytoextraction” whereby contaminants are removed and stored within the service plants;
- “phytodegradation” and “phytovolatilization” whereby contaminants are degraded through chemical process within the plant or transpiration into the atmosphere;
- and “rhizodegardation” whereby microorganisms associated with plants (e.g. bacteria around their roots) “feed” on and chemically transform contaminants.^3^

The last is associated with another, newer, form of phytoremeditation that employs microplants and bacteria to create designer “biofilms” that can serve as nano-ecobots. Not long ago such nontechnology was just the stuff of theory (see Lampton 1993 and Crandall 1996 for speculative applications for ecology). Indeed, even in his 2004 textbook, *Ecological Engineering*, Patrick Kangas optimistically speculated that: “[t]here are probably many possible uses of nanotechnology in ecological engineering […] but this kind of design must wait for future developments in the field” (p. 312). Even at that time biofilms were often seen as a threat to ecological function and ecosystem health, as the algal plants and associated bacteria can cause many problems in aquatic ecosystems (e.g. eutrophication). More recent years have seen the development of many biofilm systems as ecobots that can be crucially useful for removing contaminants, such as oil and other industrial pollutants, from water and other environments via the development and applications of biofilm growth-matrixes and designer biofilm circuits (see Hegde et al. 2011; Kardel et al. 2015; Elliott et al. 2017). In essence, these biofilm systems use autonomous rhizodegradation process enacted by bacteria and enabled by microplant substrate along with strategically designed growing surfaces and autonomous biofilm growth to serve positive ecological functions by chemically transforming pollutants and producing biomass that various organisms can eat.

Another, more complex, kind of ecobot incorporates various phytoremediation and filtration processes. These go by the name “living machines,” after the pioneering set of trademarked ecobot technologies developed by John Todd (see Todd 1991; Todd et al. 1994). Todd’s living machines utilize sequences of tanks that water can pass through; where each tank contains a different kind of ecosystem that treats pollutants and waste in water in different ways. The different internal ecosystems develop autonomously through varied seeding techniques used in each tank; and they adapt, or self-organize and reorganize, their compositions relative to changing water chemistry. Todd’s original living machines where built as floating “arks” for treating lake systems (cf. Kangas 2004, Chaps. 2 and 8). However, many ecobots of this type can be and have been developed, and can incorporate other technologies to varying degrees. For example, such ecobots might use solar powered locomotion or chemotaxically directed navigation, and could also be used as vehicles for sensors that could log data remotely for research purposes (see Past Ecological Engineering Projects). Again, the possible permutations here seem endless.

Finally, there are hybrid ecobots that integrate living machines and central computer-controlled feedback systems to comprise robots that can adaptively respond to changing environmental variables. Interestingly these comparatively complex and high tech ecobots have been discussed for quite a while in ecological engineering. Clark et al. (1999) called them “ecocyborgs” (after Parrott 1996), which they define as ‘systems that contain biological and technological components interacting as an ecosystem’. Even earlier, Odum (1993) considered ecobots calling them “technoecosystems,” in which he says, “formerly wild components of ecosystems are incorporated into technological systems as hybrids.” The notable difference between these definitions is that the former sees hybrid ecobots as formerly “natural” systems that are given some sort of artificial centralized controls (and are thereby “cyborged”) while the later sees ecobots as ecosystems that are functionally intertwined with artificial systems. The proposed conception of ecobot is therefore different, in that it sees the ecobot designation as a functional one; since the proposed conception has ecobots as *ecologically functional* robots. Accordingly, the proposed conception encompasses what have formerly been called ecocyborgs as well as what have been called technoecosystems. What are such things exactly? Again, examples help illustrate what they can be.

Although other, more rudimentary, examples exist (see, e.g., Myers and Clark 1944), the hybrid ecobots along the lines of what was just described implement autonomous feedbacks to control overall system functionality via control of key ecological processes through the integration of computer systems. A good example is John Peterson’s integration of an artificial feedback loop into an aquatic ecosystem using dissolved oxygen sensors, UV-lights, and a data-logging computer (see Petersen 2001). In this ecobot, the computer turned lights on to stimulate photosynthesis when dissolved oxygen level got too low for optimal algal growth and the resulting dissolved oxygen turned off the lights again when it measured too high for optimal system conditions. Other examples have also used computer systems and sensors for system-functioning indicators to build in similar engineered, yet autonomous, feedbacks. For instance, Cai et al. (2006) developed a similar ecobot with feedbacks triggered by pH levels and Blersch (2010) recounts the developing and testing of his “artificially intelligent biosystem” that uses functional indicators to control feedbacks that maintain biofilm growth rates in aquatic systems for water quality remediation.

Notably, each of these experimental hybrid ecobots were self-contained and monitored closely in lab and field experiments. Yet, it is easy to imagine more highly autonomous applications via the incorporation of such hybrid ecobot technologies into solar or wind-powered “living machines” as described above. On can also easily imagine how such hybrid systems could be scaled up, and could serve as subsystems in larger and more complex interlinked autonomous hybrid ecobots. Today an entire forest or a lake or a city could quite easily become a large hybrid ecobot with many smaller subsystems.

### Anticipating an Ethics of Environmental Robotics

Each of the examples of different sorts of environmental robots discussed above carry out different functions (e.g. observation, sample collection, bio-mimicking, and remediation) in different ways; and yet all are embedded within initiatives to protect and advance values associated with environmental resources. There is some prima facie intuitive pull toward assuming that all of these applications should by fiat be considered innovative and positive. Indeed, if a company were to indicate that they were ‘investing in environmental robotics,’ the public perception would likely be positive and people may be excited and eager to purchase the products of said company without understanding what that could really mean. Of course, it is important to recognize that each of the kinds of environmental robots discussed pose ethical considerations and potential risks that must be addressed alongside their potential positives. As just one very simple example, consider the use of drones for monitoring endangered species, and the ethical issue of creating stress on some species as a result of the drone’s presence. This could help guide when it might be appropriate to use drones for such research and when it may not. Such questions also bear on design considerations since drones can sometimes have a negligible impact on organism stress just by being camouflaged (e.g. by being made to look like a swan).

The number of possible novel ethical and practical issues explodes once one considers the variation in capabilities, levels of autonomy, and potential in situ impacts of each kind of environmental robot. The proposed taxonomy can help organize and sort through these issues, but it is admittedly only part of the conceptual machinery needed to effectively evaluate ethical and practical challenges raised by environmental robotics. The next step is attempting to devise an ethical framework that is sensitive to the issues associated with each kind of environmental robot. We believe that trying to reconcile prior work in the fields of environmental ethics and robot ethics is a promising initial step for trying to devise such a framework.

#### A Look to Environmental and Robot Ethics

Environmental ethics deals with the questions concerning human responsibility toward the environment in terms of: whether or not such a responsibility exists; where the justification for any such responsibilities stems from; and what such responsibilities demands of us in terms of taking or refraining from concrete actions. Justifications for responsibilities toward the environment may be grounded in the instrumental value the environment has for various human purposes and wellbeing (known as anthropocentrism) or the value the environment has on its own without necessarily being for the benefit of humans (known as bio or eco-centrism).

Robot ethics is a relatively new discipline that focuses on ethical issues resulting from the introduction of robots into various aspects of human life and how they may impact quality of life and well-being (Capurro 2009; Lin et al. 2011; Sullins 2011, Sharkey 2008). Robot ethics is both prospective and retrospective; it deals with the various life phases of the robot, i.e. the design phase, production phase, use phase, and removal phase (van Wynsberghe 2016). Robot ethicists have proposed three dimensions of importance to reveal the range of ethical issues; the ethics related to the people designing the robots, the ethics of the people using the robots, and the ethics of the robots themselves (Asaro 2006). Thus, a full ethical analysis of environmental robotics must address the various life phases of the robot in question; the multiple actors who bear responsibility for the robot’s impact; the various environmental and stakeholder values that may come into play at each step; and considerations of tradeoffs between anthropocentric and eco-centric goods.

There are then multiple ways of approaching the tangle of ethical issues environmental robotics may pose. In the tradition of applied ethics, one might use a particular ethical theory to evaluate any kind of robot’s impact in different scenarios (e.g. consequentialism, deontology, virtue ethics, and so on). Evaluating their impact could also be done in a variety of ways, for example looking at the ways in which robots could offset environmental problems or one could address the impact that robots have on the environment. For the former, robots could be used to help target: pollution, exhaustion of both renewable and non-renewable resources, or degradation of the environment (e.g. a decline in biodiversity). For the latter, robots could enhance the negative effects of the above mentioned problems and/or create new ones. And, as is common in contemporary environmental ethics, one could examine ways in which any kind of robots may enhance or degrade the various values of any environmental resource.^4^ In addition to these “applied” approaches, one can also simply bring attention to possible issues of concern to begin a debate on how to best address them. This paper will now conclude by doing just that; identifying key ethical issues related to the different kinds of environmental robots identified above. However, four brief caveats are in order regarding the discussion that follows.

First, the following discussion forgoes rigorous ethical reflection and takes no stance on one ethical theory over another to use in the evaluation, as that will be the task of future work. This paper’s central aim is more to provoke debate about the various ethical concerns concerning environmental robots and to provide resources for more effectively examining such concerns head on in follow-up work. In support of this aim of facilitating further exploration of issues surrounding such robots, the follow discussion concentrates on simply outlining some unique issues that will need to be addressed to develop best practices for their design, use, and regulation.

Second, one should take note that there are ethical considerations related to robotics writ large that are not explicitly addressed in this paper, as these issues are not unique to robots-in-ecology or ecobots. Such considerations include (among others): the environmental impact of the type of materials used to make the robots; the environmental impact of the degradation process when the robot is no longer in use; the process for testing the robot; and the amount and kind of evidence to show the robot meets its goal.

Third, ethical issues are only broadly outlined for robots-in-ecology and ecobots only. This is a simple matter of economy; since all robots-for-ecology are simply specialized robots-in-ecology (see Fig. 1 above).

Finally, please notice that the following discussion assumes that ethical issues for designers and developers are separable from those for potential end-users. This assumption is made to broadly orient considerations of responsibility in the development and uses of environmental robots, since those designing and producing robots are often not the same individuals (or groups) using them. This is true in the case of academics and NGOs conducting ecological research using a purchased off-the-shelf robot. It would be unfair to insist that end-users be responsible for the development process in which they played no part, and on some scores equally unfair to insist that designers be responsible for ethical considerations related to the eventual uses of their robots. Thus, the proceeding discussion assumes that users of environmental robots will often have different ethical considerations from those designing and producing robots and vice versa.^5^

#### Ethical Issues Concerning Robots-in-Ecology

One can begin to see novel ethical issues for robots-in-ecology by contrasting them with other technologies that are used for observing and monitoring species. Traditional biologging technologies—‘the use of miniaturized animal-attached tags for logging data’ for instance–are a good example of a comparable technology (Rutz and Hays 2009). Robots used to observe and monitor can also provide data like more standard biologging techniques, and run into similar ethical issues regarding potential physiological stresses they may cause some species. Robots-in-ecology differ from standard tagging and biologging applications, however, in that their presence has been shown to have advantages in some cases.

For instance, it was noted above that they permit less invasive monitoring of certain bird species. And notably their very presence has been shown to lower the instance of poaching in pilot studies of specific use cases by upwards of 96% in some cases (Choi-Fitzpatrick 2014). Another thing that is unique to such robots-in-ecology is the potential that the robot can be hacked and the information used by malicious users. If the robot were used to collect images or data of human users (as is the case when using drones in humanitarian contexts), this could raise concerns about privacy of the data and informed consent of participants; for example, robots used for monitoring are also capable of capturing information of nearby human subjects. So, such technologies may present concerns about data provenance and data protection that simpler technologies do not.

In the case of monitoring with robots, one can also already see “secondary” uses that were not intended at the original time of data collection. One might speculate, for example, that the monitoring of species migratory patterns could provide insight about the impacts of climate change on those and other species. If such unanticipated conclusions are borne out by novel research enabled by robots like UAVs, this opens up questions about whether the salient researchers have a responsibility to share the new information and implications of it. A similar type of situation occurs in healthcare when individuals participate in experiments and/or trials and new information (e.g. about a disease or pre-existing condition) is revealed. Surely, researchers have a responsibility to deal with these issues with great care, and many questions arise regarding what they may have a responsibility to share.^6^ Here we simply want to flag that uses of robots-in-ecology have the potential to present new concerns in this vein about the responsibilities of their users.

Of course, a larger class of ethical concerns center on the potential for various harms that robots-in-ecology may present. Already noted above, is that, along with their potential for minimizing stress, the presence of certain technologies can also cause physiological stresses to certain species. Such technologies can also malfunction and cause harms that might otherwise not occur (e.g. UAVs crash on occasion). These potentials for causing harm via research are not unique to robots-in ecology, but their consideration does direct one to concerns about their being designed ethically. For example, aesthetic considerations like those discussed above may shift in light of such concerns. If, for instance, it proves that a robot resembling aspects of an environment or species in it decreases potential stresses its presence would cause otherwise, this may imply that there is a corresponding responsibility on the part of users to choose robots that will minimize stress to the species in the intended environment (appearance, materials, and so forth). Accordingly, certain key ethical considerations for robots-in-ecology are directed mainly towards potential users of such robots; including considerations of: impacts on the observed species; the security of data collection; and the (secondary) uses of the data collected.

#### Ethical Issues Concerning Ecobots

When considering ecobots, there appears to be a closer link between the ethical issues raised for users and those raised for designers, because such robots will likely most often not be re-purposed off-the-shelf robots but specifically designed for a particular context and use. In the event that the user is also the designer (as in the case with Todd and the not-for-profit RSE mentioned above) mitigating risks seems more achievable. In the event that the user is not also the designer, collaboration between the two is surely advisable to minimize and/or mitigate the concerns raised here.

That being said, there are issues specifically concerning the user, and/or the use, of any ecobot. To begin, one may question whether or not such a technology constitutes tampering with nature, is it justified, and furthermore how to systematically approach such justifications. A central issue presented by robots-for-ecology regards responsibilities for their maintenance and continued management of the ecosystems they are placed in. Could the failure or removal of such a robot weaken the system if said system was relying on the robot to fulfill a role/function? Is it possible that the robot will become requisite for some systems to flourish? If the latter is true then one might suggest that users of the robot have a responsibility to maintain and manage the robot’s presence indefinitely. Consider, as a parallel, cases in which robots are used for human stroke rehabilitation in hospital contexts and the human user is saddened or otherwise negatively impacted by the removal of the robot after the testing phase (Hughes et al. 2014).

We would also echo the abovementioned design and aesthetic concerns. For ecobots, it seems that designers may have design responsibilities that bear great weight in certain cases; considering that such robots will be delegated functional roles in ecological and sociecological systems into which they will be integrated and integral. Neglecting such concerns could easily constitute a maleficence or neglect on the part of the designer of such systems or on the part of users who implement ecobots without considering the ramifications these design features may have. As such, it is advisable to develop best practices and policies for the design and use of ecobots to ensure that such considerations are addressed or at the very least considered.

Other design issues related to ecobots concern their composition and design. There are concerns about the use of inorganic materials introduced into an ecological systems glossed above but it is potentially of greater weight to raise concerns over the introduction of engineered organic materials into a natural environment. Considering that such things as biofilms, and even certain biomimicking robots, can be capable of altering their form or functionality without direct human input, the possible constitutions and materials used for making certain ecobots adds special kinds of unpredictability to the systems in which they may be embedded.

Even more worrying concerns about human user(s) losing control of ecobots placed in an ecosystem come up when considering hybrid ecobots, “ecocyborgs,” that integrate computers. As the main technological disciplines related to robotics converge, AI is a driving force for robotics and machine learning and big data are the driving forces for AI. Thus, it is possible that some such ecobots could gain in capabilities (through machine learning, AI, and big data feedbacks), and could quickly achieve a level of autonomy so as to no longer need humans in the loop for decision-making. Hence, in the same way that the ethics of AI deals with concerns over the unpredictability and transparency of system outputs, the ethics of environmental robotics must confront pressing questions regarding the different sorts of autonomy exercised by hybrid ecobots. Further concerns about losing control of hybrid ecobots combine some of the abovementioned issues since they too may present risks due to hacking. Finally, it is noteworthy that even those ecologists who dreamt up such ecobots saw potentials for serious risks associated with hybrid systems becoming larger, more complex, and more and more a part of human’s environments. As Clark et al. (1999) note in their treatment of hybrid ecobots, as “ecocyborgs,” it is easily “possible that such ecocyborgs would simply be too large and complicated to be effectively controlled […b]y humans” (p. 3).^7^

## Conclusion

This paper is in no way intended to dissuade against the development or use of environmental robots nor to condemn those involved in their development and use. On the contrary, its authors are optimistic about the use of robots for the benefit of environmental research and protection—especially considering the current global political climate and the fact of inevitable unprecedented environmental changes due to the human influence on Earth systems. It appears that robots will be a driving force for the future global economy and the trend of increased uses of robotics in environmental research and engineering will continue. With these things in mind, we urge that ensuring that environmental robotics proceeds in a responsible manner—one which pays tribute to values associated with the environment and environmental resources—requires developing and implementing best practices for both research and industry applications of environmental robotics. Toward that end, we have sought to hasten the dawning of the ethics of environmental robotics by: explaining and taxonomizing the kinds of environmental robots that exist to date; and suggesting avenues for further explorations of their potential valuable applications, and the ethical, practical, and sociopolitical issues they may present. In so doing, we have provided resources we hope will facilitate and guide more pointed analyses of the potential challenges that emerging environmental robot technologies may present.

## Footnotes

1 An associated incentive is found in the potential of the expansion of ecological robotics to help grow and sustain the movement toward a green economy. In the same way that renewable energy sources like wind and solar will create jobs—estimated to be a $6 trillion industry by 2030 (see Disgraceful Exit)—while mitigating the use of environmental hazards, robotics can do the same.

2 The survey of robots-in-ecology here is admittedly coarse and by no means exhaustive (see Dunbabin and Marques 2012 and Grémillet et al. 2012 for history and additional categorization).

3 This list is far from exhaustive; cf. Green and Hoffnagle (2004); Chappell (1997).

4 Though it must be defended elsewhere for lack of space, we believe that contemporary virtue ethics approaches to environmental ethics are rich with resources for developing an ethics of environmental robots. Such views naturally reconcile environmental and robot ethics, as well as anthro and eco-centric considerations, through the ideas ‘external goods,’ including ecological integrity and functionality, that can promote “flourishing” (Shockley 2014, pp. 209–210).

5 This is not to say that this separation in responsibilities is ideal or that things will remain like this in the future. Quite the opposite in fact, we would rather see a future in which developers worked closely with end users to anticipate and mitigate negative outcomes earlier in the design process.

6 Data collected from environmental robots could also be valuable for political or business endeavors; as can be seen observe with biologging data, which “are increasingly being applied to management and conservation policy (Peckham et al 2007; cf. Burger and Shaffer 2008).

7 This is not to suggest that large autonomous hybrid ecobots will be analogous to the “autonomous thinking machines” of Bostrum (Bostrum & Yudkowsky 2004) or the AI of Stephan Hawking that will take over the world; but we do believe consideration of ethical issues regarding the various sorts of autonomy environmental robots can pose require serious attention. The future of autonomous robots generally will most likely rely on AI to increase levels of autonomy and the AI algorithms will most often rely on big data for their training. And environmental robots that are useful for applications considered too dangerous for humans, or in areas that are impossible for humans to reach, make the development of more highly autonomous robots very appealing (see Marques 2012 and Siegwart et al 2004). So, with the inevitability of increased production and uses for environmental research and protection, comes the inevitably of more and more highly autonomous robots-in-ecology and ecobots.
